# CBT—Intervention for panic disorder in primary care: 5 years follow-up of a cRCT during the Covid-19 pandemic

**DOI:** 10.1371/journal.pone.0287718

**Published:** 2023-06-30

**Authors:** Karoline Lukaschek, Carolin Haas, André Wannemüller, Christian Brettschneider, Tobias Dreischulte, Jürgen Margraf, Jochen Gensichen, on behalf of the PARADIES study group

**Affiliations:** 1 Institute of General Practice and Family Medicine, University Hospital, LMU Munich, Munich, Germany; 2 Graduate Program “POKAL—Predictors and Outcomes in Primary Care Depression Care” (DFG-GrK 2621), Munich, Germany; 3 Mental Health Research and Treatment Center, Ruhr-University Bochum, Bochum, Germany; 4 Department of Health Economics and Health Services Research, Hamburg Center for Health Economics, University Medical Center Hamburg-Eppendorf, Hamburg, Germany; 5 Institute of General Practice and Family Medicine, Jena University Hospital, Jena, Germany; Edith Cowan University, AUSTRALIA

## Abstract

A practice team-based exercise programme with elements of cognitive behavioural therapy (CBT) and case management for patients with panic disorder with or without agoraphobia in primary care showed significant positive effects. Here, we analyse the long-term effects (>5 years) of this intervention in the stressful context of the Covid-19 pandemic. All participants of the original PARADIES cluster randomized controlled trial (cRCT; 2012–2016) were invited to participate in a follow-up during the Covid-19 pandemic. Clinical outcomes were anxiety symptoms, number and severity of panic attacks, agoraphobic avoidance behaviour, Covid-specific anxiety symptom severity, depression, and patient assessment of chronic illness care. Data were analysed cross-sectionally for group differences (intervention, control) and longitudinally (T_0_: baseline, T_1_: 6 months and T_Corona_: >60 months). Of the original 419 participants, 100 participated in the 60 months follow-up (October 2020-May 2021). In the cross-sectional analysis, the anxiety symptom severity in the intervention group was lower than in the control group (p = .011, Cohen‘s d = .517). In the longitudinal analysis, both groups showed an increase of anxiety and depression symptoms compared to pre-pandemic level. The intervention may have had a lasting impact regarding anxiety severity despite the challenging context of the Covid-19 pandemic. However, we cannot say to what extend the intervention still played a role in participants’ lives; other factors may also have helped with coping. The increase of anxiety and depression symptoms in both groups over time could be attributed to external circumstances.

## Introduction

The Global Burden of Disease (GBD) estimated that the COVID-19 pandemic has led to a 25.6% increase (95% uncertainty interval (UI): 23.2–28.0) in cases of anxiety disorders worldwide in 2020 [1]. It is estimated to have caused 116.1 (95% UI: 79.3–163.80) additional DALYs (disability-adjusted life years) per 100 000 population [2]. Recently, three meta-analyses, involving ~50,000 individuals compared levels of self-reported mental health problems during the COVID-19 pandemic with those before the pandemic, and reported a small increase in anxiety symptoms with pooled effect sizes between 0.13–0.17 [3]. Many people who had previously coped well with their anxiety symptoms had difficulties in coping with the pandemic-related stress factors (e.g. fear of infection, loneliness, social distancing). Individuals with pre-existing mental disorders may be at increased risk for worsening anxiety symptoms during the pandemic [4]. For this vulnerable group, it can be aggravating that (mental) health care was not accessible at the height of the Covid-19 pandemic [3].

Panic disorder is an anxiety disorder characterized by reoccurring unexpected panic attacks. Agoraphobia refers to avoidance or endurance with dread of situations from which escape might be difficult or help unavailable in the event of a panic attack. Typical agoraphobic situations include shopping malls, theatres, traveling by bus, crowded restaurants, and being alone [5]. The International Classification of Diseases (ICD)-10 classifies panic disorder with or without agoraphobia as F41.0 or F40.01.

Cognitive behavioural therapy (CBT) shows strong effectiveness for a variety of mental illnesses, including anxiety-related disorders [6, 7]. Several randomised controlled trials have shown that CBT works well in the treatment of panic disorder with or without agoraphobia [7–9], even when provided by a non-specialist (e.g. general practitioner, nurse, medical assistant) [10].

The PARADIES (“Patient Activation foR Anxiety DIsordErS”) trial, a two-armed cluster randomized controlled trial (cRCT), was conducted between 2012 and 2016 in the German federal states of Bavaria, Hesse, and Thuringia in rural or urban general practitioners (GPs) practices [8]. Patients with panic disorder with or without agoraphobia allocated to an intervention practice received case management and CBT-based therapy provided by the GP. In the intervention group, symptoms of anxiety improved to a significantly greater extent (p = 0.008), and there was a significantly greater reduction in the frequency of panic attacks (p = 0.019), in avoidance behaviour (p = 0.016), and depression (p<0.001).

There are few studies only that follow up the effectiveness of CBT for patients with anxiety disorders over a longer period of time (> 2 years) [11–14]. Despite their heterogeneity, the overall results of these few studies point to the long-term effectiveness of CBT (e.g. effect sizes ranging from 0.31 to 0.92 for depression and anxiety related disorders [11]). One meta-analysis supports an association with CBT and improved outcomes of anxiety related disorders until 12 months after treatment completion [7].

The aim of the present study was to investigate whether the positive effects of a practice team-based intervention ("PARADIES") with elements of CBT for primary care patients with panic disorder with or without agoraphobia (ICD-10: F41.0 or F40.01) still hold up five years after its end in the context of a natural crisis such as the Covid-19 pandemic. For this reason, we assessed anxiety and depression related clinical parameters in a sample of the original study population in order to measure potential long-term success.

## Methods

### Original study design

The aim of the PARADIES cluster randomized trial (cRCT; conducted between 2012 and 2016) was to deliver a low intensity and effective therapy to people with panic disorder with or without agoraphobia in a primary care setting. This cRCT in 73 GPs’ practices included 419 patients with panic disorder with or without agoraphobia (mean age: 46.2 years [standard deviation: 14.4]; 74% female). At baseline, patients were blinded to group membership. Patients in the intervention group (IG, 36 practices, 230 patients) received case-management [15], practice team-supported exposure training and four appointments with GP including evidence-based elements of CBT (psychoeducation, interoceptive and situational anxiety exposure exercises, relapse-prevention) [8, 16]. Patients in the control group (CG, 37 practices, 189 patients) were treated according to guideline-based standard therapy [17, 18].

Initially, patients were screened by participating GPs and their teams, using the Overall Anxiety Severity and Impairment Scale (OASIS) [19] and the panic modules of the Patient Health Questionnaire (PHQ) [20]. After that, diagnosis was confirmed by the GP’s diagnostic interview following validated ICD-10 check lists. Only adult patients with a diagnosis of panic disorder with or without agoraphobia (ICD-10: F41.0 or F40.01) were included. Exclusion criteria were: suicidality, psychotic or substance-related disorders, severe physical impairment, pregnancy or current anxiety-specific psychotherapy.

The PARADIES study was approved by Ethics Committee of the Friedrich-Schiller University Jena on 17 August 2012 (no. 3484–06/12). All participating physicians and patients gave their written informed consent to participating in the study. The study was registered with Current Controlled Trials (ISRCTN64669297) and the German Clinical Trials Register (Deutsches Register Klinischer Studien, DRKS, DRKS00004386).

### Present study sample

**Fig 1:**
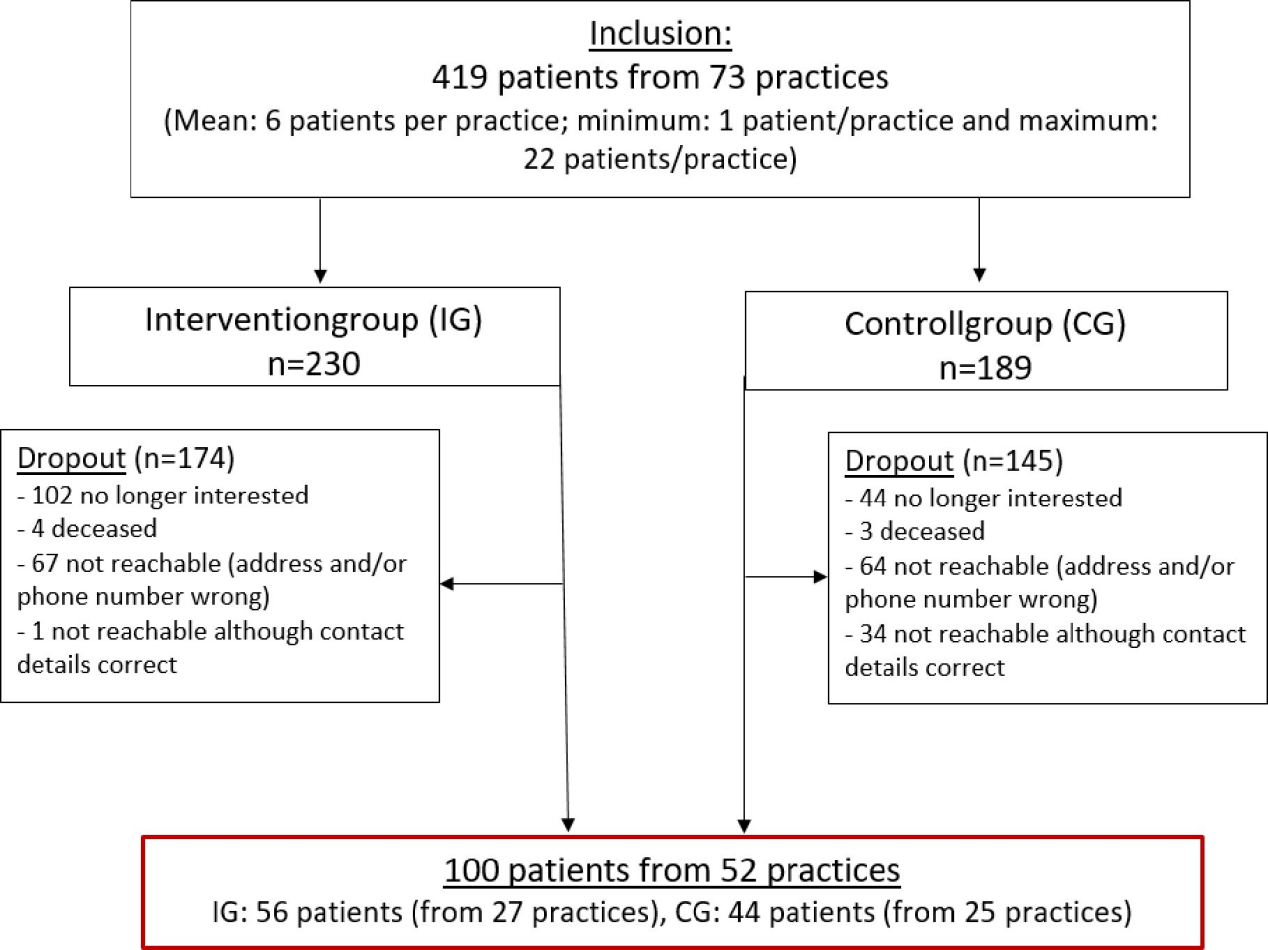
Flow chart.

For the 60 months follow-up, all patients (N = 419) of the original PARADIES cRCT (conducted between 2012 and 2016) were contacted repeatedly in various ways (telephone, letter, email) from October 2020 to May 2021. The follow-up was completed by 56/230 (24%) patients from 27/36 practices in the IG and 44/189 (23%) patients from 25/37 practices in the CG. In sum, a total of 100 patients from 52 practices could be included in the present study. For a detailed description of participants and drop-outs, see flow chart (Fig 1). Patients’ characteristics are stated in Table 1.

**Table 1:** Characteristics of patients.

	Intervention group		Control group	
**Patients, n (%)** *	56 (56)		44 (44)	
**Mean age in years (± SD)**	57,4 (11,6)		53,8 (11,5)	
**Female sex, n (%)**	42 (75,0)		34 (77,3)	
**Living in a partnership, n (%)**	42 (75,0)		36 (81,0)	
**Outpatient treatment in the last 6 months**	**Patient N**	**Average days**	**Patient N**	**Average days**
•Psychiatrist	5	12	10	26
•Psychologist/psychotherapist	12	79	12	95
•General practitioner, family doctor	37	185	32	133

*Patients’ characteristics of the original study see supplement and original publication [8].

### Clinical outcomes

The severity of anxiety was assessed with the Beck Anxiety Inventory (BAI) [21]. Patients were asked how much they were burdened by 21 typical anxiety symptoms in the past week (range: 0–63. Higher scores indicate greater severity of clinical anxiety: 0–7 minimal anxiety; 8–15 mild anxiety; 16–25 moderate anxiety; 26–63 clinically relevant anxiety). The BAI has demonstrated high internal consistency (Cronbach’s alpha = .94) [22]; its validity and sensitivity to change have also been demonstrated for the primary medical setting [23]. The overall severity and functional impairment caused by anxiety symptoms were measured with The Overall Anxiety Severity and Impairment Scale (OASIS, five questions, range: 0–25. Higher scores indicate higher anxiety levels and greater clinical impairment; Cronbach’s alpha = .89) [19]. Number and severity of panic attacks were measured with two items (A1, A2) of the Panic and Agoraphobia Scale (PAS,range: 1–5. Higher values for more frequent panic attacks, or more intense panic attacks, respectively; Internal consistency: Cronbach’s alpha = .90) [24]. Agoraphobic avoidance behaviour was measured with the Mobility Inventory (MIA; Cronbach’s alpha = .96), "alone" subscale (range: 1–5; higher values indicate stronger agoraphobic avoidance behaviour) [25].Depression was measured with the Patient Health Questionnaire, depression subscale (PHQ-9, range: 0–27. Higher values indicate more severe depressive symptoms; Cronbach’s alpha = .89) [20]. Patient assessment of medical care was measured with the Patient Assessment of Chronic Illness Care (PACIC, range: 1–11. Higher values indicate better patient assessment of chronic disease care; Cronbach’s alpha = .87) [26]; Corona-related anxiety was measured with the Fear-of-Covid-19 scale (range: 5–35. Higher values indicate a higher anxiety level; Cronbach’s alpha = .86). The Fear-of-Covid-19-Scale asks to what extent people feel uncomfortable or nervous when they think about Covid-19, or get physical sensations such as clammy hands or palpitations, cannot sleep because of worry, or are even afraid of dying from the virus [27].

### Statistical analysis

#### A. Cross sectional analysis of the primary and secondary outcome variables at 60 months follow-up

Descriptive analyses of the impact of the intervention on the outcome variables BAI, OASIS, MIA, PHQ-9, PAS A1, PAS A2, fear-of-COVID-19-scale and PACIC were performed with at t-test for independent samples; a p-value smaller than 0.05 meant statistical significance.

#### B. Longitudinal analysis: Comparison at baseline, 6 months follow-up and 60 months follow-up

For the comparison of three different measurement dates T_0_ (baseline), T_1_ (6 months) and T_Corona_ (≥60months follow up), the data sets are merged and checked for missing values. Since the analysis revealed missing values > 10%, no imputation was carried out and all incomplete data (in terms of individual subjects) were filtered out of the data set. In line with Shapiro-Wilk test, the residuals were not normally distributed. According to the Levene test, the homogeneity of the error variances between the groups is not given even after Box-Cox power transformation, which means that the requirement for a mixed model analysis is not met. Instead, the questionnaire values (dependent variables) were examined individually for group differences and differences over time. Bonferroni post-hoc test were performed. For the group differences, the non-parametric and distribution-free Mann-Whitney-U test was calculated for two independent samples. In order to analyse the differences between the three measurement points, a non-parametric Friedmann test for dependent samples was calculated in each case. The values of the BAI (n = 56), PHQ (n = 74), PAS frequency (n = 75) & intensity (n = 84) and the PACIC (n = 32) are included in the analysis. With regard to the MIA data, after excluding all subjects with missing values, only n = 11 remain and therefore no further analysis is done. The Kolmogorov-Smirnov-test (KST) was used to test the equality of probability distributions of the intervention group and the control group. No long-term analysis could be performed for the OASIS, as it was only used during the 60 months follow-up and comparative values for the other measurement dates (T0, T1) were missing.

All statistical analyses were carried out with IBM SPSS Statistics, version 28.0.

## Results

### A. Cross sectional analysis of the primary and secondary outcome variables at 60 months follow-up

Table 1 shows the socio-economic characteristics of the respondents. The majority of respondents were female (IG: n = 42, 75.0%; CG: n = 34, 77.3%). Overall, the mean age of respondents was 55.8 years (range: 27–81 years, SD: 11.7 years), although patients in the IG were slightly older with a mean age of 57.4 years (range: 28–81 years, SD: 11.6 years). The mean BAI scores in both groups indicated moderate severity of anxiety (IG: 16.3, CG: 19.6) (see Table 2).

**Table 2:** Cross sectional comparison at 60 months follow-up of mean sum scores in anxiety and depression symptoms.

	Intervention group (n = 56)	Control group (n = 44)	p-value
**Mean Sum Score at Corona follow-up** *			
BAI (±SD)	16,3 (12,5)	19,6 (12,9)	,215
OASIS (±SD)	7,2 (4,5)	9,6 (4,5)	**,011**
MIA (±SD)	1,95 (0,90)	2,21 (0,86)	,152
PHQ-9 (±SD)	7,0 (5,2)	8,7 (5,5)	,143
PACIC (±SD)	4,37 (3,45)	3,83 (2,81)	,466
Fear-of-COVID-19-scale (±SD)	17,00 (7,792)	18,26 (6,120)	,394
PAS_A1 (±SD)	1,7 (0,83)	1,9 (0,98)	,228
PAS_A2 (±SD)	1,8 (0,91)	2,0 (1,1)	,277

BAI, Beck Anxiety Inventory; MIA, Mobility Inventory, subscale alone; OASIS, Overall Anxiety and Impairment Scale; PACIC, Patient Assessment of Chronic Illness Care; PHQ-9, Patient Health Questionnaire; PAS, Panic and Agoraphobia Scale; SD, Standard deviation

* Missing data: BAI—4 patients from IG, 2 patients from CG; OASIS—1 patient from IG, 2 patients from CG; MIA—3 patients from IG, 1 patient from CG; PHQ-9–5 patients from IG, 2 patients CG; fear-of-COVID-19-scale: from 5 patients from IG, 5 patients from CG; PAS_A1–3 patients from IG, 2 patients from CG; PAS—3 patients from IG, 2 patients from CG.

The results of the scores measuring depression (PHQ-9), satisfaction with outpatient treatment (PACIC), frequency and severity of panic attacks (PAS_A1 and PAS_A2), the Mobility Inventory (MIA) and mean Covid-19 anxiety (fear-of-COVID-19-scale) were also better in the IG than in the CG (see Table 2). However, at 60 months follow-up, only the difference in the OASIS sum score between the groups reached nominal significance level (p = .011) with medium effect size (Cohen`s d = .517). In particular, the OASIS item inquiring to which extent anxiety symptoms interfered with work, school or homewas found to have medium effect size (Cohen`s d = .717). However, the OASIS did not reach significance level after Bonferroni-correction.

### B. Longitudinal analysis: Comparison at baseline, 6 months follow-up and 60 months follow-up

#### B1. Anxiety symptoms (BAI)

**Fig 2:**
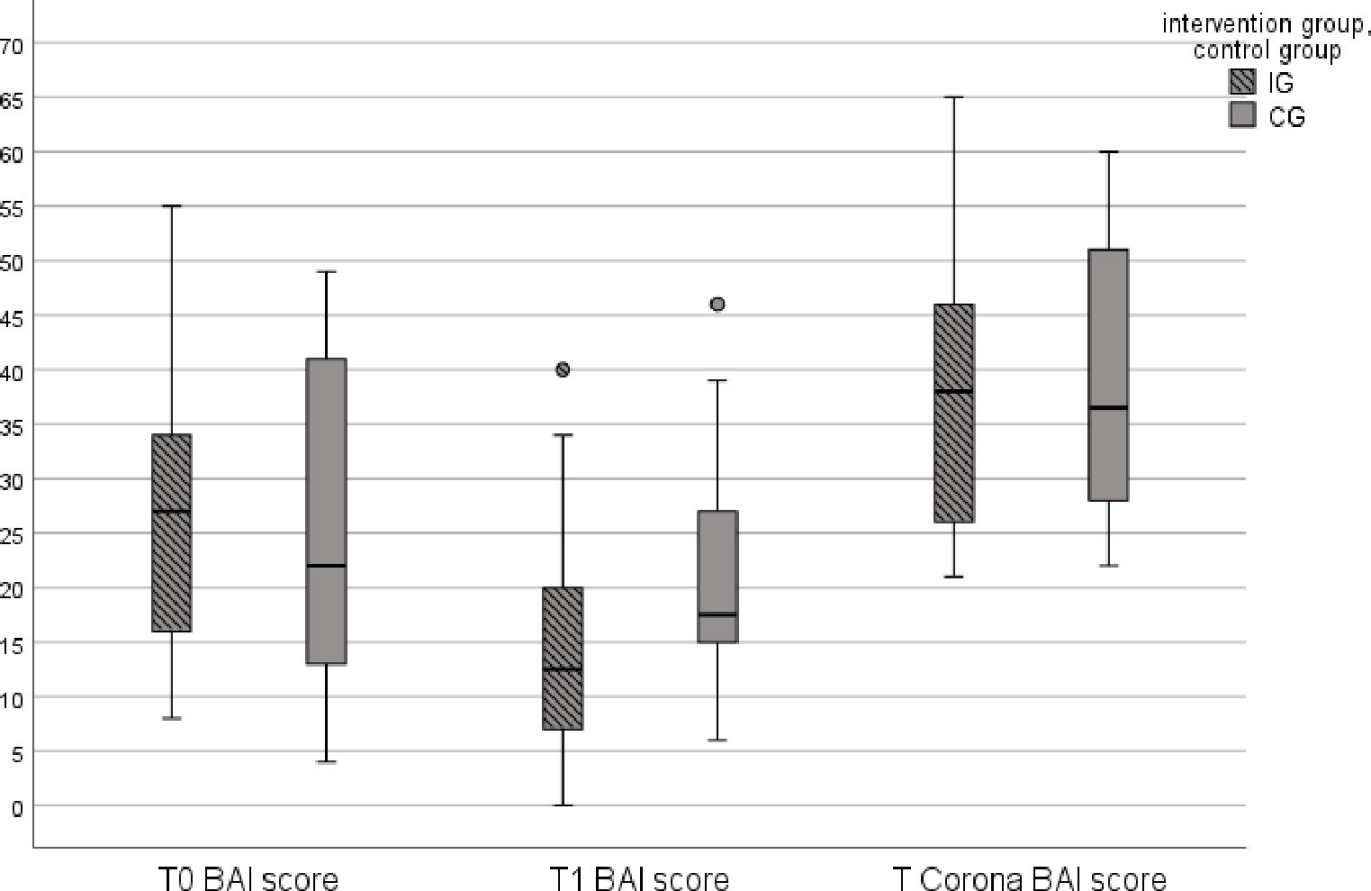
Comparison of IG and CG boxplots of median BAI score at different time points (T0, T1, T_Corona_).

The KST (p > .05) indicated equal distributions for IG (n = 34) and CG (n = 22). There was a statistically significant difference in median BAI anxiety symptoms at T_1_ between IGn (Mdn = 12.50) and CG (Mdn = 17.50), U = 234, Z = -2.351, p = .019. However, there was no statistically significant difference in BAI median anxiety symptoms at the 60 months follow up between both groups, U = 329.5, Z = -.747, p = .455. Fig 2 shows the comparison between IG and CG boxplots of median BAI anxiety score across all three measurement dates T_0_ (IG Mdn = 27; CG Mdn = 22), T_1_ (IG Mdn = 12.5; CG Mdn = 17.5) and T_Corona_ (IG Mdn = 38; CG Mdn = 36.5). Friedman test was conducted to determine whether BAI mean rank anxiety symptoms differ between baseline (T_0_, M_Rank_ = 1.96), 6 months follow-up (T_1_, M_Rank_ = 1.35), and Corona follow-up (T_Corona_, M_Rank_ = 2.70). The results show significant differences, χ2(2) = 51.291, p < .001. A Bonferroni post-hoc test also revealed significant differences over time for each pairwise comparison: From baseline measurement to T1 anxiety symptoms (BAI) tended to decrease (T_0_-T_1_: Z = .607, p < .001, r = .081), whereas from T1 to follow up (T_Corona_), BAI Score increased again (T_1_-T_Corona_: Z = -1.348, p < .001, r = .184). Baseline to T_Corona_ also differed with an increasing tendency (T_0_-T_Corona_: Z = -.741, p = .004, r = .099.) All referred changes showed small effect sizes (r < 0.3).

#### B2. Depression symptoms (PHQ-9)

**Fig 3:**
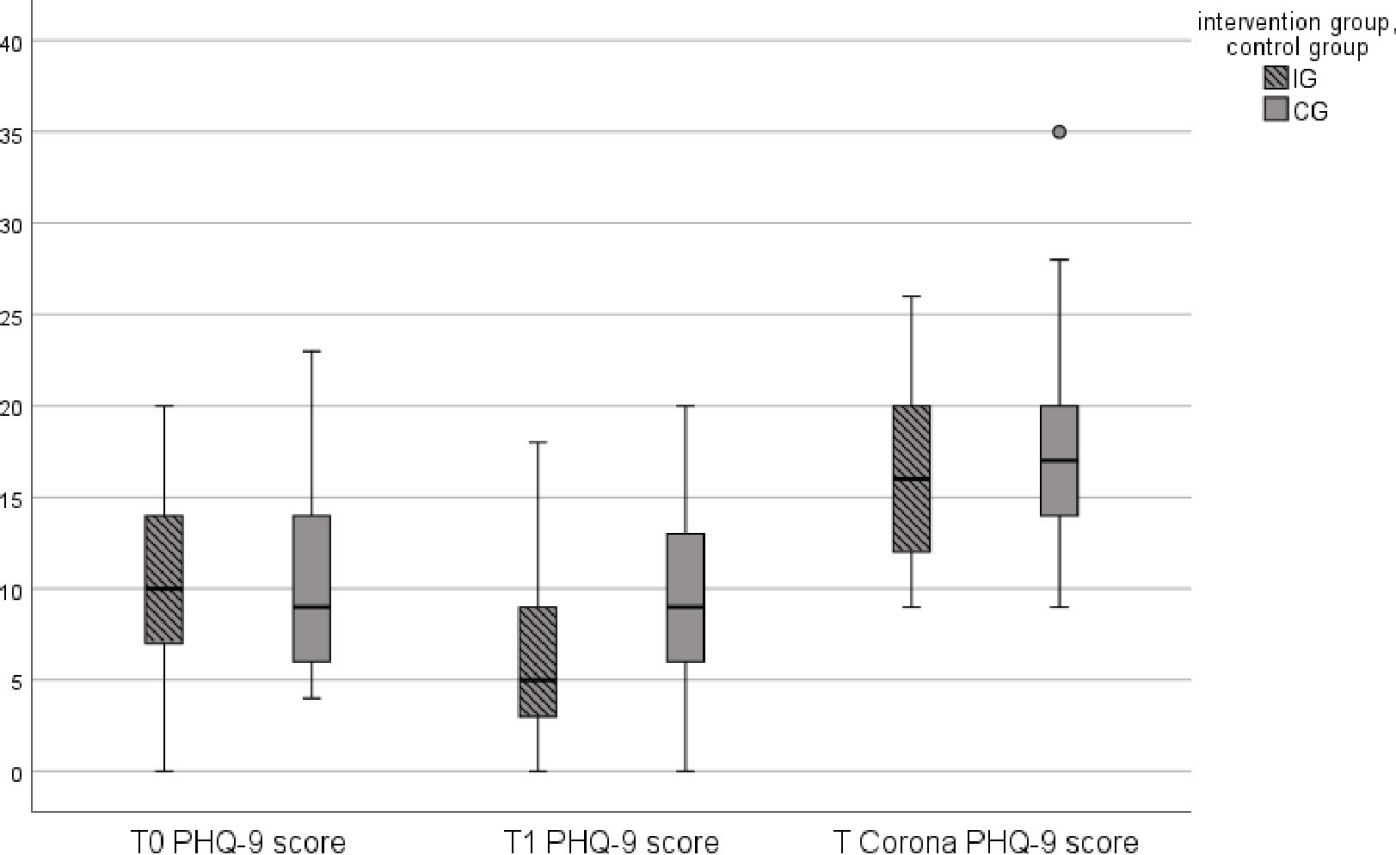
Comparison of IG and CG boxplots of median BAI score at different time points (T0, T1, T_Corona_).

The KST (*p* < .05) indicated unequal distributions for IG (n = 37) and CG (n = 37). There was a statistically significant difference in mean rank PHQ depression symptoms after six months (T_1_) between IG (M_Rank_ = 31.36) and CG (M_Rank_ = 43.64), U = 457, Z = -2.461, p = .014. However, there was no statistically significant difference in mean rank PHQ depression symptoms at the 60 months follow-up (T_Corona_) between both groups, U = 585.5, Z = -1.072, p = .284. Fig 3 shows the comparison between IG and CG boxplots of median PHQ depression score across all three measurement dates T_0_ (IG Mdn = 10; CG Mdn = 9), T_1_ (IG Mdn = 5; CG Mdn = 9) and T_Corona_ (IG Mdn = 16; CG Mdn = 17). Friedman test was conducted to determine whether PHQ mean rank depression symptoms differ between baseline (T_0_, M_Rank_ = 1.83), 6 months follow-up (T_1_, M_Rank_ = 1.43), and 60 months follow-up (T_Corona,_ M_Rank_ = 2.74). The results show significant differences, χ2(2) = 68.089, p < .001. A Bonferroni post-hoc test also revealed significant differences over time for each pairwise comparison: From baseline measurement to T1 depressiv symptoms according to PHQ-9 tended to decrease (T_0_-T_1_: Z = .405, p = .014, r = .047), whereas from T1 to follow up (T_Corona_), PHQ-9 Score increased again (T_1_-T_Corona_: Z = -1.318, p < .001, r = .153). Baseline to T_Corona_ also showed an increasind tendency (T_0_-T_Corona_: Z = -.912, p < .001, r = .106) All referred changes showed small effect sizes (r < .3).

#### B3. Frequency of panic attacks (PAS)

(*p* < .05) indicated unequal distributions for IG (n = 38) and CG (n = 37). There was a statistically significant difference in mean rank PAS frequency item after 6 months (T_1_) between IG (M_Rank_ = 30.39) and CG (M_Rank_ = 45.81), U = 414, Z = -3.261, p = .001. However, there was no statistically significant difference in mean rank PAS frequency of panic attacks at the 60 months follow-up between both groups, U = 622.5, Z = -.916, p = .360. Friedman test was conducted to determine whether PAS mean rank frequency of panic attacks differ between baseline (T_0_, M_Rank_ = 2.04), 6 months follow-up (T_1_, M_Rank_ = 1.62), and 60 months follow-up (T_Corona_, M_Rank_ = 2.34). The results show significant differences, χ2(2) = 23.927, p < .001. A Bonferroni post-hoc test revealed significant differences over time for pairwise comparison between T_0_ & T_1_ as well as T_1_ & T_Corona:_ From baseline measurement to T1 frequency of panic attacks (PAS) tended to decrease (T_0_-T_1_: Z = -.420, p = .010, r = .048.), whereas from T1 to follow up (T_Corona_), PAS-frequency-score increased again (T_1_-T_Corona_: Z = .720, p < .001, r = .083). All referred changes showed small effect sizes (r < .3). No changes could be reported from baseline to T_Corona_ (T_0_-T_Corona_: Z = .300, p = .066).

#### B4. Intensity of panic attacks (PAS)

The KST (*p* < .05) indicated unequal distributions for IG (n = 45) and CG (n = 39). There was a statistically significant difference in mean rank PAS intensity item after 6 months (T1) between IG (MRank = 37.48) and CG (MRank = 48.29), U = 651.5 Z = -2.131, p = .033. However, there was no statistically significant difference in mean rank PAS intensity of panic attacks at the 60 months follow-up between both groups, U = 834.5, Z = -.417, p = 676. Friedman test was conducted to determine whether PAS mean rank intensity of panic attacks differ between baseline (T_0_, MRank = 2.05), 6 months follow-up (T_1_, MRank = 1.63), and 60 months follow-up (T_Corona_, MRank = 2.32). The results show significant differences, χ2(2) = 24.577, p < .001. A Bonferroni post-hoc test revealed significant differences over time for pairwise comparison between T_0_ & T_Corona_ as well as T_1_ & T_Corona_: There were no changes from baseline measurement to T1 regarding the intensity of panic attacks according to PAS (T_0_-T_1_: Z = .262, p = .090). From baseline measurement to follow up (T_Corona_) as well as from T1 to follow up (T_Corona_), PAS-intensity-score increased (T_1_-T_Corona_: Z = .423, p = .006, r = .046; T_0_-T_Corona_ Z = .685, p < .001, r = .075). All referred changes showed small effect sizes (r < .3).

#### B5. Patient Assessment of Chronic Illness Care (PACIC)

The KST (*p* < .05) indicated unequal distributions for IG (n = 14) and CG (n = 18). There was neither a statistically significant difference in mean rank PACIC after 6 months (T_1_) between IG (M_Rank_ = 17.47) and CG (M_Rank_ = 15.25), U = 86, Z = -1.521, p = .135, nor was there a statistically significant difference in mean rank PACIC at the 60 months follow-up between both groups, U = 119.5, Z = -.258, p = .808. Friedman test was conducted to determine whether PACIC mean rank differ between baseline (T_0_, M_Rank_ = 2.19), 6 months follow-up (T_1_, M_Rank_ = 2.55), and 60 months follow-up (T_Corona,_ M_Rank_ = 1.27). The results show significant differences, χ2(2) = 28.173, p < .001. A Bonferroni post-hoc test revealed significant differences over time for pairwise comparison between T_0_ & T_Corona_ as well as T_1_ & T_Corona_: There were no changes in PACIC from baseline measurement to T1 (T_0_-T_1_: Z = -.359, p = .151). From baseline measurement to follow up (T_Corona_) as well as from T1 to follow up (T_Corona_), PACIC-score decreased (T_1_-T_Corona_: Z = 1.281, p < .001, r = .226; T_0_-T_Corona_ Z = .922 p < .001, r = .163). All referred changes showed small effect sizes (r < .3).

## Discussion

More than five years after the original trial ended, we investigated potential long-term effects of a practice team-based intervention (CBT and case management) for patients with panic disorder during the Covid-19 pandemic. Group differences of the anxiety severity (OASIS) in cross sectional analysis at the 60months follow-up were found with medium effect sizes. Especially one item differed between groups with large effect size (“How much does anxiety or fear affect your ability to complete necessary tasks at work, school, or home?”), indicating that the intervention group suffered less from impairment through fear. However, these are very tentative interpretations, since after Bonferroni correction significance level was no longer reached.

In general, while CBT is widely recognised as effective treatment for panic disorders [28–30], evidence is scarce that CBT can be associated with a better outcome regrading panic disorder after 12 or more months of follow up [7, 13]. Yet the effects of psychotherapy can remain at a high level for a long time (up to 2 years) after treatment ends; however, relapse may occur again after years. Naturalistic follow-up observations over several years suggest that there is a significant recurrence of symptoms years after the termination of cognitive-behavioral therapy [18]. Panic disorder is associated with poor functioning, reduced health-related quality of life, and more sick days at work [31]. A meta-analysis that investigated the influence of cognitive-behavioral therapy (CBT) on quality of life conducted a subanalysis based on different domains of quality of life (i.e., physical, psychological, social, and environmental domains): Improvements resulting from CBT were greater in the physical and psychological domains compared to the environmental and social domains. Extensive CBT interventions had a greater effect on quality of life than brief interventions [32].

Interestingly, in our study, the IG and CG differed moderately to strongly with regard to anxiety severity and impairment (measured by OASIS) but not with regard to the other anxiety scales (measured by BAI, PAS, Fear-of-Covid-19-Scale, and MIA). This might be related to what the different anxiety scales address in detail: The BAI asks about various physiological anxiety symptoms, the two PAS items address frequency & intensity of panic attacks, Fear-of-Covid-19-Scale refers to fears and physical sensations in association with the virus and the MIA deals with agoraphobic avoidance behaviour. The OASIS uniquely asks specifically how fear impairs people in their everyday life: The last two OASIS items examine the extent of anxiety-related impairments in professional/domestic (Item 4) and social (Item 5) domains and the total sum score negatively correlates with the construct of perceived quality of life [33].

We assume that CBT-based interventions including psychoeducation may have a positively affected patients’ understanding of their symptoms; thus, they might feel less impaired by their panic attacks and anxiety symptoms in everyday life, even in a crisis situation like the Covid-19 pandemic. Furthermore, the pandemic might have resulted in positive and potentially buffering changes for some, including a better work–life balance through home-office [3].

In the longitudinal analysis, IG and CG both showed an increase in anxiety and depression symptoms, which might be attributed to the external circumstances brought about by the pandemic. The slightly better values of the IG regarding anxiety and depression could be due to the fact that these patients might have maintained behaviour and self-management strategies, which helped them cope with the crisis situation. However, we must also keep in mind that there might have been many other factors within the six years after the end of the original trial which influenced people’s behaviour and which we did not assess.

Germany-wide data from surveys conducted by a large insurance company over the last 30 years showed that in the German population, anxiety levels were at an all-time low in 2020 and 2021 [34]. However, in 2022 anxiety levels rose again due to fears related to the Ukrainian war and rising living costs.

Patient assessment of chronic care was significantly lower during the 60 months follow-up, probably because GP care reached its limit during the pandemic: GPs were overburdened with vaccinations and Covid-19 patients; additionally, they had to cope with staff shortages due to the Covid-19 pandemic. Thus, they might not have the appropriate time for their chronic patients. Our findings coincide with a survey that was commissioned by the AOK-Bundesverband (Federal Association of the health insurance “AOK”) and has been conducted in several waves since 2019 (N = 2000 participants). It found that since the beginning of the Covid-19 pandemic, fewer and fewer Germans believe that health care in their region works well or very well [35].

During the Covid-19 pandemic, access to health services was difficult; thus, technological advancements, e.g. eHealth supported tools or iCBT, should be considered as promising solutions [36].

## Strength and limitations

The strength of the present analysis is its thorough assessment of anxiety and depression outcomes during a natural crisis situation. A limitation of the study is the low response rate. Although we tried several ways to contact participants, we could only include participants who were accessible and willing to participate. Small numbers of subjects may result in lower statistical power. Thus, we want to emphasize that the interpretations of our results is tentative.

Moreover, it is possible that only particularly motivated and psychologically less stressed–or conversely, particularly stressed–patients participated. We tried to counteract potential selection bias by repeatedly contacting reluctant participants. Compared to the original trial, participants at follow-up were older than the elapsed time would suggest. This may be why the online questionnaire was so poorly received. We tried to address this through phone calls and postal letters. In our study, anxiety severity (OASIS) showed a difference between IG and CG. However, due to the cross-sectional character of the OASIS-data, we cannot draw firm conclusions regarding the long-term effectiveness of a practice team-based CBT-intervention in primary care. The PARADIES intervention is not part of standard care in Germany and thus, only available from few GPs.

## Conclusion

A practice team-based intervention (CBT and case management) for primary care patients with panic disorder with or without agoraphobia may have had a lasting impact regarding anxiety severity despite the challenging context of the Covid-19 pandemic. CBT strategies which patients can apply on their own after termination of treatment (e.g. exposure therapy, behavioural activation, reduction of avoidance behaviour) seem to be a promising strategy for promoting long-term success. However, other factors may have also helped with coping, and therefore, it is unclear to what extent the intervention still played a role in participants’ lives. Additionally, the increase in anxiety and depression symptoms in both groups over time could be attributed to external circumstances, highlighting the need for further research to better understand the impact of the pandemic on mental health.
